# A Hybridization of Dragonfly Algorithm Optimization and Angle Modulation Mechanism for 0-1 Knapsack Problems

**DOI:** 10.3390/e23050598

**Published:** 2021-05-12

**Authors:** Lin Wang, Ronghua Shi, Jian Dong

**Affiliations:** School of Computer Science and Engineering Central South University, Changsha 410083, China; csuwanglin@csu.edu.cn (L.W.); shirh@csu.edu.cn (R.S.)

**Keywords:** angle modulation mechanism, trigonometric generating function, dragonfly algorithm, binary optimization, 0-1 knapsack problem

## Abstract

The dragonfly algorithm (DA) is a new intelligent algorithm based on the theory of dragonfly foraging and evading predators. DA exhibits excellent performance in solving multimodal continuous functions and engineering problems. To make this algorithm work in the binary space, this paper introduces an angle modulation mechanism on DA (called AMDA) to generate bit strings, that is, to give alternative solutions to binary problems, and uses DA to optimize the coefficients of the trigonometric function. Further, to improve the algorithm stability and convergence speed, an improved AMDA, called IAMDA, is proposed by adding one more coefficient to adjust the vertical displacement of the cosine part of the original generating function. To test the performance of IAMDA and AMDA, 12 zero-one knapsack problems are considered along with 13 classic benchmark functions. Experimental results prove that IAMDA has a superior convergence speed and solution quality as compared to other algorithms.

## 1. Introduction

Being some of the most important and widely used algorithms, gradient-based traditional optimization algorithms are relatively mature and have advantages like high computational efficiency and strong reliability. However, traditional optimization methods have critical limitations when applied to complex and difficult optimization problems because (i) they often require that the objective function is convex, continuous and differentiable and the feasible region is a convex set, and (ii) their ability to process non-deterministic information is poor.

Over the years, plenty of algorithms based on artificial intelligence, sociality of biological swarms, or the laws of natural phenomena have emerged and been proved to be good alternative tools for solving such complex problems. This type of optimization algorithms can be roughly divided into the following five categories: (i) Evolutionary algorithms (EAs); (ii) swarm intelligence; (iii) simulated annealing [1]; (iv) tabu search [2,3]; and (v) neural networks. EAs include genetic algorithms (GA) [4,5], differential evolution [6], and immune system [7]. Among these three algorithms, GA is based on the concept of survival of the fittest mentioned in Darwin’s theory of evolution. GA and DE can be considered as the most standard form of EAs. The swarm intelligence algorithms include classic particle swarm optimization (PSO) [8], bat algorithm [9], artificial bee colony [10], ant colony algorithm [11], firefly algorithm [12], artificial fish-swarm algorithm [13], fruit fly optimization algorithm [14], and so on. These algorithms mentioned above are based on social activities of birds, bats, honey bees, ants, fireflies, fish, and fruit flies, respectively. They are far less perfect in theory than the traditional optimization algorithms at present, and often fail to ensure the optimality of the solution. However, considering the perspective of practical applications, this kind of budding algorithms generally do not require the continuity and convexity of the objective function and constraints, and they also have excellent ability to adapt to data uncertainty.

The dragonfly algorithm (DA) is a new swarm intelligence optimization algorithm that was proposed by Mirjalili [15] in 2015. It is inspired by two unique clusters of dragonflies found in nature: Foraging groups (also known as static groups) and migratory groups (also known as dynamic groups). These two group behaviors of dragonflies are very similar to the two terms of group intelligence (global search and local development). In the static group, dragonflies will be divided into several sub-dragonfly groups to fly in different areas, which is the main target of the global search. In the dynamic group, dragonflies will gather into a large group and fly in one direction, which is advantageous for the local development. Since the principle of DA is simple, easy to implement, and possesses good optimization capabilities, it has shown promising results when applied to multi-objective optimization [15], image segmentation problem [16], and parameter optimization of support vector machines [17]. Moreover, DA has also been successfully applied to the accurate prediction model of power load [18], power system voltage stability evaluation [19], power flow management of smart grid system [20], economic dispatch [21], synthesis of concentric circular antenna arrays [22], and traveling salesman problem [23]. Further, based on a large number of numerical tests, Mirjalili proved that DA performs better than GA [4,5] and PSO [8].

It must be noted that DA was used to solve the continuous optimization problem, while many optimization problems have existed in binary search spaces. This suggests that the continuous version of the optimization algorithm can no longer meet the requirements of the binary optimization problems. A binary version of DA(BDA) was proposed by Mirjalili et al. [15] and successfully applied to the feature selection problems [24]. Like binary PSO (BPSO) [25] and binary BA [26], BDA used a transfer function to map the continuous search space into binary space. In [27], Hammouri et al. proposed three improved versions of BDA, named Linear-BDA, Quadratic-BDA, and Sinusoidal-BDA, for feature selection. By using different strategies to update main coefficients of the dragonfly algorithm, the three algorithms outperform the original BDA. However, such binary algorithms were still developed by using transfer functions, which may be limited in some high-dimensional optimization problems owing to slow convergence speed and poor algorithm stability.

To avoid such problems, intelligent optimization algorithms based on the angle modulation technique, originated in signal processing [28], were proposed recently such as angle modulated PSO [29], angle modulated DE [30], and angle modulated bat algorithm [31]. Inspired by these algorithms, an angle modulated dragonfly algorithm (AMDA) is proposed in this paper to make DA work more efficiently in binary-valued optimization spaces. By using a trigonometric function with four coefficients to generate *n*-dimensional bit strings, AMDA is observed from the experiments on benchmark functions and 0-1 knapsack problems to have better performance as compared to other optimization algorithms such as BPSO and BDA. Further, by adding a control coefficient to adjust the vertical displacement of the cosine part of the generating function, an improved angle modulated dragonfly algorithm (IAMDA) is proposed to enhance convergence performance and algorithm stability.

The rest of this paper is arranged as follows. The standard DA and the binary DA (BDA) are elaborated in Section 2. In Section 3, the proposed AMDA and IAMDA are explained. Further, Section 4 presents the analysis of the experimental results on 13 benchmark test functions and 12 0-1 knapsack problems. Finally, Section 5 discusses and concludes the performance of IAMDA with respect to BPSO, BDA, and AMDA.

## 2. Background

The dragonfly algorithm (DA) is a budding algorithm inspired by the social behavior of dragonflies, and this section gives a brief introduction about DA and its binary version.

### 2.1. The Dragonfly Algorithm

**Figure 1:**
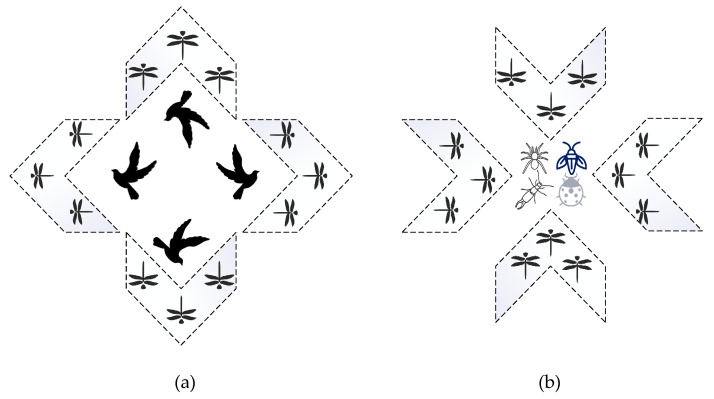
Dynamic swarms (**a**) versus static swarms (**b**).

The dragonfly algorithm is an advanced swarm-based algorithm inspired by the static and dynamic clustering behaviors of dragonflies in nature. By simulating the behaviors of dragonflies looking for prey, mathematical modeling of the algorithm is done. During the modeling, the life habits of dragonflies, such as finding food, avoiding natural enemies, and choosing the flight routes are considered. The dragonfly population is divided into two groups: migratory swarm (also known as a dynamic swarm) and feeding swarm (also known as a static swarm). A large number of dragonfly clusters migrate in a common orientation for long distances intending to seek a better living environment in the dynamic swarm whereas, in a static swarm, each group is composed of a small group of dragonflies that fly back and forth in a small area to find other flying prey. The migration and feeding behaviors of dragonflies can be regarded as two main phases in meta-heuristics algorithm optimization: exploitation and exploration. Dragonflies gather into a large group and fly in one direction in a dynamic swarm, which is beneficial in the exploitation phase. In a static swarm, however, to find other flying prey, small groups of dragonflies fly back and forth in a small range, which is beneficial to the exploration of search agents. The dynamic and static groups of dragonflies proposed by Mirjalili [15] are demonstrated in Figure 1.

Separation, alignment, and cohesion are three main principles in the insect swarms introduced by Reynolds [32] in 1987. The degree of separation refers to the static collision avoidance of the individuals from other individuals in the neighborhood, the degree of alignment indicates the velocity matching of individuals to that of other individuals in the neighborhood, and the degree of cohesion reflects the tendency of individuals toward the center of the mass of the neighborhood.

Every swarm in DA follows the principle of survival, and each dragonfly exhibits two separate behaviors: looking for food and avoiding the enemies in the surrounding. The positioning movement of dragonflies consists of the following five behaviors:

(1) Separation. The separation between two adjacent dragonflies is calculated as follows:

(1) $$S_{i}=-\sum_{j=1}^{N}(X_{i}-X_{j})$$

where $S_{i}$ is the separation of the *i-th* individual, $X_{i}$ is the location of the *i-th* individual, $X_{j}$ indicates the location of the *j-th* neighboring individual, and *N* is the number of neighborhoods.

(2) Alignment. The alignment of dragonflies is calculated as follows:

(2) $$A_{i}=\frac{\sum_{j=1}^{N}V_{j}}{N}$$

where $A_{i}$ indicates the alignment of *i-th* individual, $V_{j}$ indicates the velocity of the *j-th* neighboring individual, and *N* is the number of neighborhoods.

(3) Cohesion. The cohesion is derived as follows:

(3) $$C_{i}=\frac{\sum_{j=1}^{N}X_{j}}{N}-X_{i}$$

where $C_{i}$ indicates the cohesion of the *i-th* individual, $X_{i}$ is the position of the *i-th* individual, *N* represents the number of neighboring individuals, and $X_{j}$ shows the location of the *j-th* neighboring individual.

(4) Attraction. The attraction toward the source of food is calculated as follows:

(4) $$F_{i}=X^{+}-X_{i}$$

where $F_{i}$ shows the food source of the *i-th* individual, $X_{i}$ indicates the location of the *i-th* individual, and $X^{+}$ represents the location of the food source. 

(5) Distraction. The distraction from an enemy is derived as follows:

(5) $$E_{i}=X^{-}+X_{i}$$

where $E_{i}$ represents the position of an enemy of the *i-th* individual, $X_{i}$ is the location of the *i-th* individual, and $X^{-}$ indicates the location of the natural enemy.

**Figure 2:**
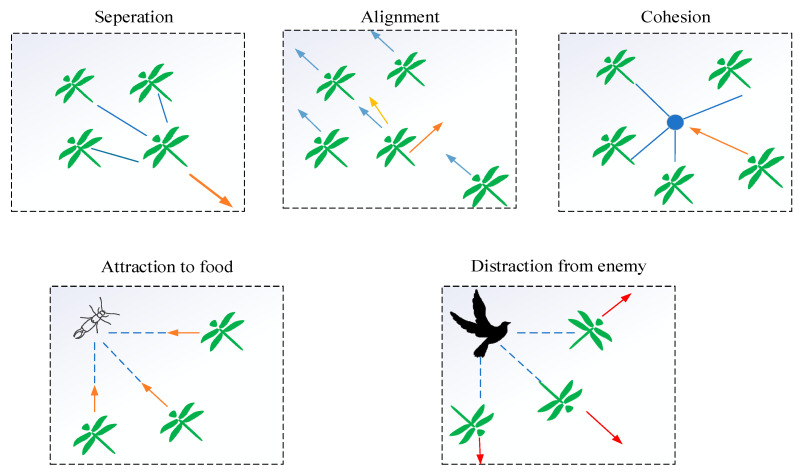
The five main social behaviors of dragonfly swarms.

The above five swarming behaviors in the positioning movement of dragonflies are pictorially demonstrated in Figure 2.

To update the location of dragonflies in a search space and to simulate their movements, two vectors are considered: step vector (Δ***X***) and position vector (***X***). The step vector suggests the direction of the movement of dragonflies and can be formally defined as follows:

(6) $$\Delta X_{i}^{t+1}=(sS_{i}+aA_{i}+cC_{i}+fF_{i}+eE_{i})+w\Delta X_{i}^{t}$$

where *s* is the separation weight, $S_{i}$ is the separation of the *i-th* individual, *a* shows the alignment weight, $A_{i}$ indicates the alignment of *i-th* individual, *c* is the cohesion weight, $C_{i}$ indicates the cohesion of the *i-th* individual, *f* represents the food factor, $F_{i}$ shows the food source of the *i-th* individual, *e* indicates the enemy factor, $E_{i}$ represents the position of an enemy of the *i-th* individual, *w* represents the inertia weight, and *t* represents the iteration count.

According to the calculation of the above step vector, the position vector can be updated by using Equation (7):

(7) $$X_{i}^{t+1}=X_{i}^{t}+\Delta X_{i}^{t+1}$$

If there are no neighboring solutions, the positon vectors are calculated by using the following equation:

(8) $$X_{i}^{t+1}=X_{i}^{t}+Levy(dim)\times X_{i}^{t}$$

where *dim* is the dimension of the position vector. Levy function can be described as follows:

(9) Levy(dim)=0.01×r1×σ|r2|1β

where *r*_1_ and *r*_2_ are random numbers within [0,1], *β* is a constant, and:

(10) σ={Γ(1+β)×sin(πβ2)Γ(1+β2)×β×2(β−1)/2}1β

where Γ(z)=(z−1)!

**Figure 3:**
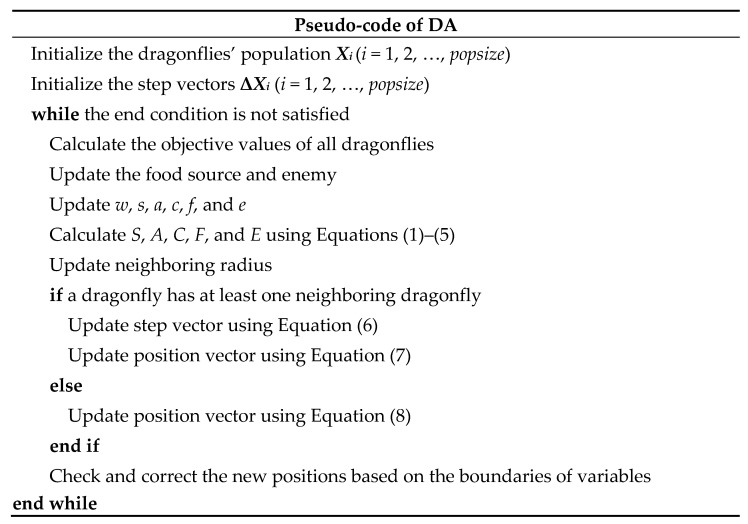
Pseudo-codes of DA.

The basic steps of DA can be summarized as the pseudo-codes highlighted in Figure 3.

### 2.2. Binary Dragonfly Algorithm

In the traditional DA, a search agent can easily change its position by introducing a step vector. However, in the discrete spaces, since a position vector can only be updated to 0 or 1, it is impossible to update a position vector according to the original method. Mirjalili et al. [15] first proposed the binary dragonfly algorithm (BDA) to solve the binary optimization problems. BDA adopted the following transfer function to derive the probability of changing positions of all the search agents:

(11) $$T(\Delta x)=\left|\frac{\Delta x}{\sqrt{\Delta x^{2}+1}}\right|$$

Further, the position vectors can be updated by the following formula:

(12) $$X_{i}^{t+1}=\{\begin{array}{c} {}^{\neg }X_{i}^{t},r\leq T(\Delta x^{{}_{t+1}})\\ X_{i}^{t},r>(\Delta x^{{}_{t+1}}) \end{array}$$

where *r* is a random number between [0,1].

## 3. Improved Angle Modulated Dragonfly Algorithm (IAMDA)

### 3.1. AMDA

In this paper, the angle modulation technique is used for the homomorphic mapping of DA to convert the complex binary optimization problem into a simpler continuous problem. Different from the traditional BDA, the angle modulated dragonfly algorithm (AMDA) uses a trigonometric function to generate bit strings. The trigonometric function can be expressed as:

(13) g(x)=sin(2π(x−a)×b×cos(2π(x−a)×c))+d

where *x* = 0, 1, …, *n_b_* − 1, denotes the regular intervals at which the generating function is sampled, where *n_b_* is the length of the required binary solution; the four coefficients (*a*, *b*, *c*, and *d*) are within [–1,1] at initialization. Then, the standard DA is used for evolving a quadruple composed of (*a*, *b*, *c*, *d*), and this leads each dragonfly to generate a position vector of the form ***X****_i_* = (*a*, *b*, *c*, *d*). To evaluate a dragonfly, the coefficients from the dragonfly’s current position are substituted into the generating function in Equation (13). Each sampled value at *x* is then mapped to a binary digit as follows:

(14) $$g(x)=\{\begin{array}{c} 0,g(x)\leq 0\\ 1,g(x)>0 \end{array}$$

**Figure 4:**
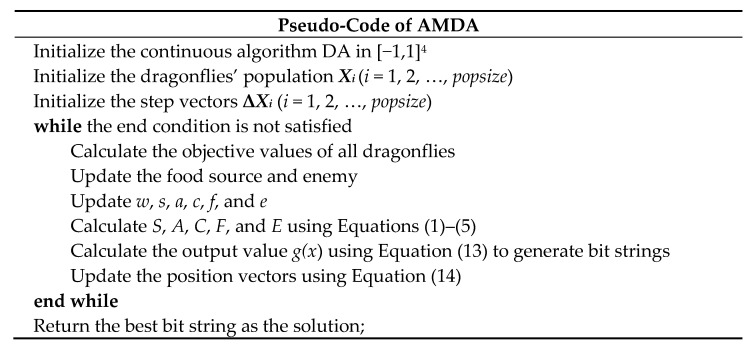
Pseudo-codes of AMDA.

The main steps of AMDA are simplified as the pseudo-code givens in Figure 4.

### 3.2. IAMDA

The prime advantage of AMDA is that it only needs four coefficients instead of the original *n*-dimensional bit strings. Thus, the computational cost will be significantly reduced. AMDA’s generating function is a composite of a sine wave and a cosine wave. The vertical displacement of the sine wave can be controlled by the coefficient *d* in Equation (13) but the vertical displacement of the cosine wave cannot be corrected, which results in a large variance of the entire generating function value. In addition, if the initialization range of DA parameters is small, DA will encounter some difficulties while searching for a binary solution.

To alleviate the problem of the inability and control the vertical displacement of the cosine wave in the original generating function, this paper proposed an improved AMDA, called IAMDA. IAMDA uses one more coefficient *k* to control the degree of disturbance of the generating function in the mapping space:

(15) g(x)=sin(2π(x−a)×b×cos(2π(x−a)×c)+k)+d

where the five coefficients (*a*, *b*, *c*, *d,* and *k*) are within [−1,1] at initialization. The standard DA is used for evolving a quintuple composed of (*a*, *b*, *c*, *d*, *k*), and this led each dragonfly to generate a position vector of the form ***X****_i_* = (*a*, *b*, *c*, *d*, *k*). To evaluate a dragonfly, the coefficients from the dragonfly’s current position are substituted into Equation (15) and each sampled value is then mapped to a binary digit according to Equation (14).

**Figure 5:**
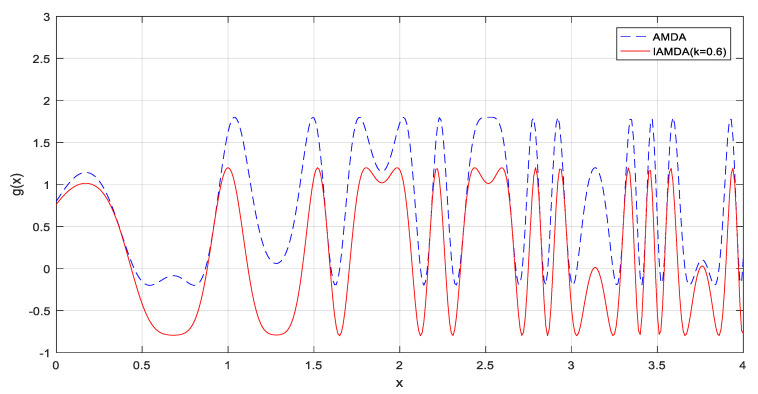
The original and modified generating functions. (*a* = 0, *b* = 0.5, *c* = 0.8, *d* = 0.2).

In the original generating function, if the value of *d* is not large enough, the generating function will always be above or below 0, which will make the bit string only contain bit 0 or 1. Hence, a coefficient *k* is added to generate a bit string containing both 0 and 1 bits. The coefficient *k* is introduced to compensate for the insufficient disturbance in trigonometric function as well as to adjust vertical displacement of the cosine function. The comparison between the original and modified generating functions is presented in Figure 5. It can be observed from Figure 5 that the original generating function with the vertical displacement *d* = 0.2 is almost above 0. In this manner, it is easier to generate solutions that are mostly 0s or 1s. In the modified generating function, the displacement coefficient *k* increases the diversity of the solutions so that IAMDA may achieve better solutions even if the vertical displacement *d* is not large enough.

**Figure 6:**
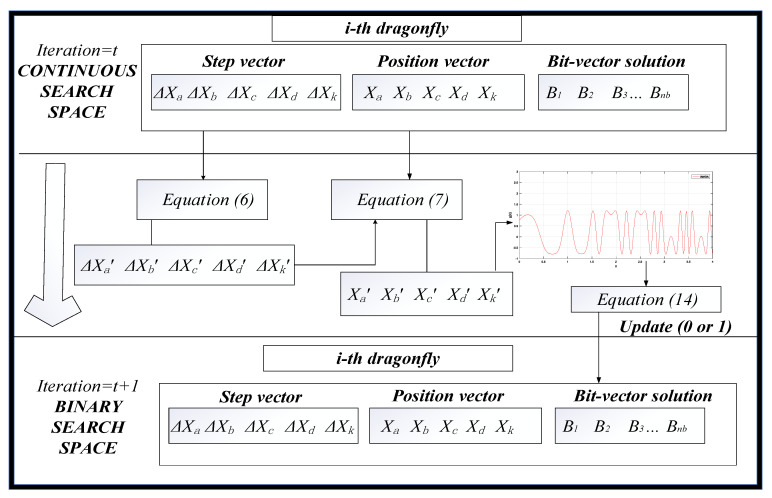
The process of mapping a continuous five-dimensional search space to an *n*-dimensional binary search space.

**Figure 7:**
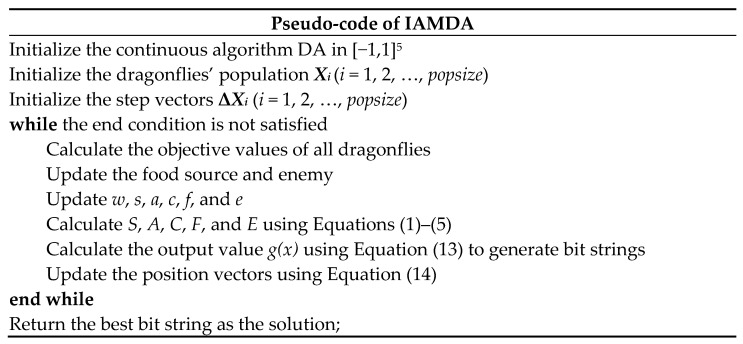
Pseudo-codes of IAMDA.

In order to demonstrate the mapping procedure, Figure 6 shows the procedure of using the modified trigonometric function to map a continuous five-dimensional search space into an *n*-dimensional binary search space. The main procedures of IAMDA are described as the following pseudo-codes given in Figure 7.

## 4. Experimental Results and Discussion

### 4.1. Test Functions and Parameter Settings

To verify the performance and stability of IAMDA, two sets of benchmark test functions and 0-1 knapsack problems are selected. To efficiently compare the performance of each algorithm, the original AMDA, the basic BDA [15], and the BPSO [25] were selected to deal with the test problems. The average solution, median, and standard deviation are taken into consideration to evaluate each algorithm.

In this paper, the population size of IAMDA, AMDA, BDA, and BPSO is set to be 30 [15,33,34] and the number of iterations is set to be 500. Other parameter settings are listed in Table 1. To avoid the resulting bias caused by chance, the algorithms run independently on each function 30 times. Moreover, in this paper, each continuous variable is represented by 15 bits in binary. It should be noted that in order to indicate the sign of each functions’ variable, one bit should be reserved. Hence, the dimension of each dragonfly, that is, the dimension of each generated bit string can be calculated as follows:

(16) $$Dim_{dragonfly}=Dim_{function}\times 15$$

where $Dim_{dragonfly}$ and $Dim_{function}$ represent the dimension of each dragonfly in IAMDA and the dimension of a specific benchmark function, respectively.

Simulation environment: The processor is an Intel(R) Core (TM) i5-6500 2.40GHz, with 4.0GB RAM, Windows10 operating system, and the simulation software is Matlab2016a.

### 4.2. IAMDA Performance on Unimodal and Multimodal Benchmark Functions

The test functions are categorized into two groups: unimodal functions (*f*_1_~*f*_7_) and multimodal functions (*f*_8_~*f*_13_) [15,35,36]. To solve the optimal function, IAMDA is compared with several other algorithms on the 13 standard test functions. Each unimodal benchmark function has a single optimal value and it is easy to benchmark the convergence speed and optimization capability of an algorithm. On the contrary, multimodal benchmark functions have multiple optimal values, which makes them more complex as compared to unimodal functions. There is only one global optimal value among many optimal values, and an algorithm ought to avoid all local optimal approximations and tend to find the global optimal value. Hence, the multimodal test function can efficiently benchmark the exploration of the algorithm and the avoidance of local optima. The specific conditions about unimodal functions as well as multimodal functions are highlighted in Table 2 and Table 3, respectively. Here, ‘Function’ indicates the test functions, ‘*n*’ represents the number of variables in the test function, ‘Range’ demonstrates the search scope of the test function, and ‘f_min_’ indicates the global optimal value of the test function.

**Figure 8:**
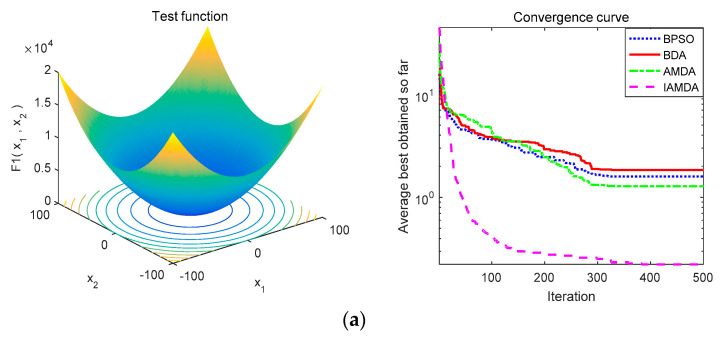

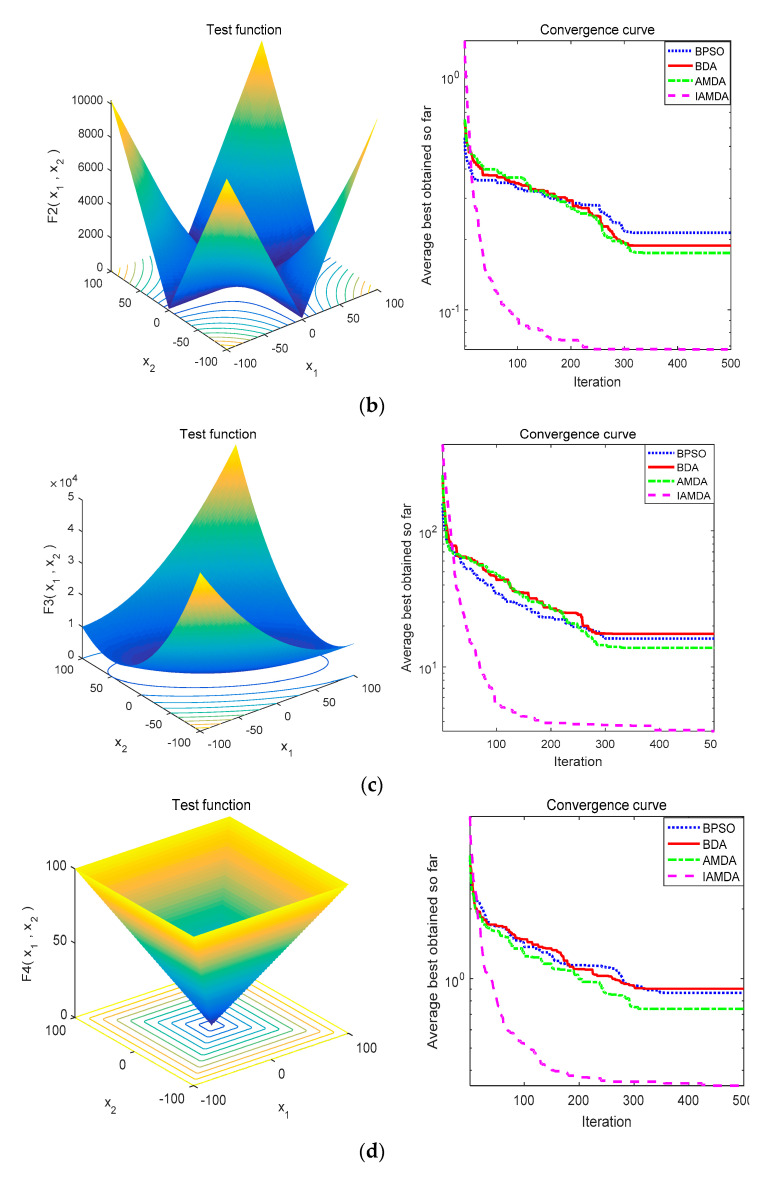

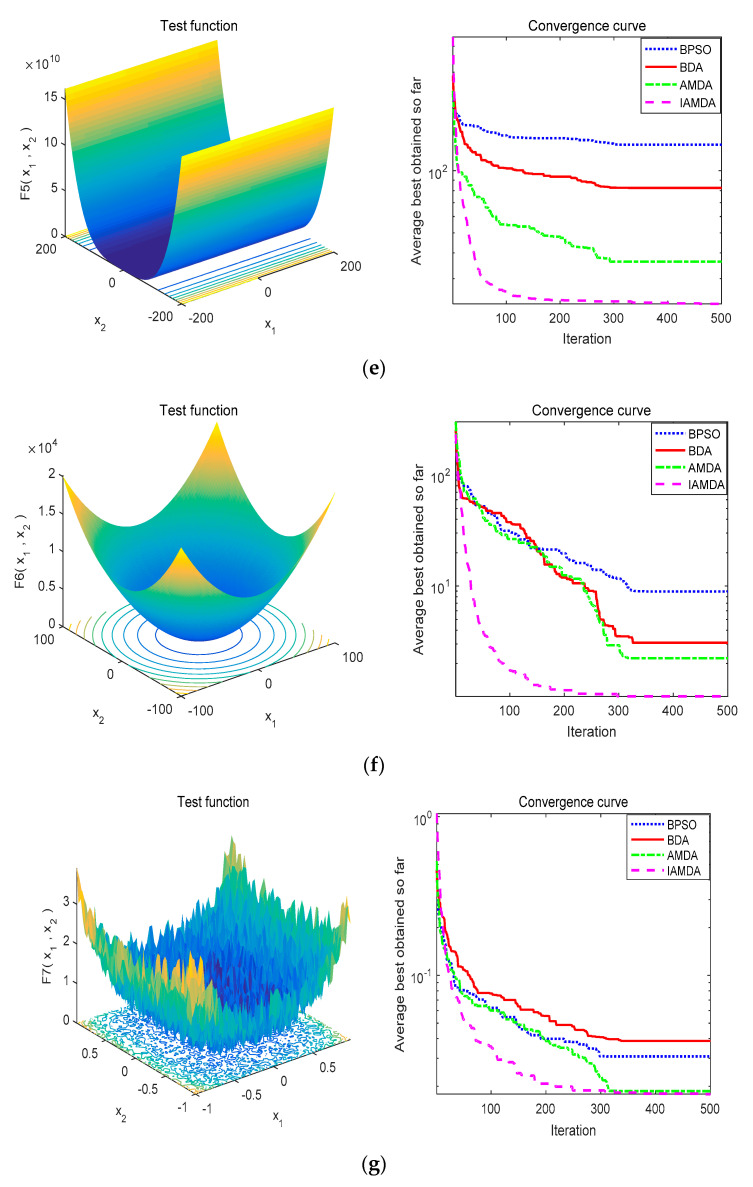
Convergence curve of IAMDA, AMDA, BDA, and BPSO on unimodal functions. (**a**) *f*_1_. (**b**) *f*_2_. (**c**) *f*_3_. (**d**) *f*_4_. (**e**) *f*_5_. (**f**) *f*_6_. (**g**) *f*_7_.

**Figure 9:**
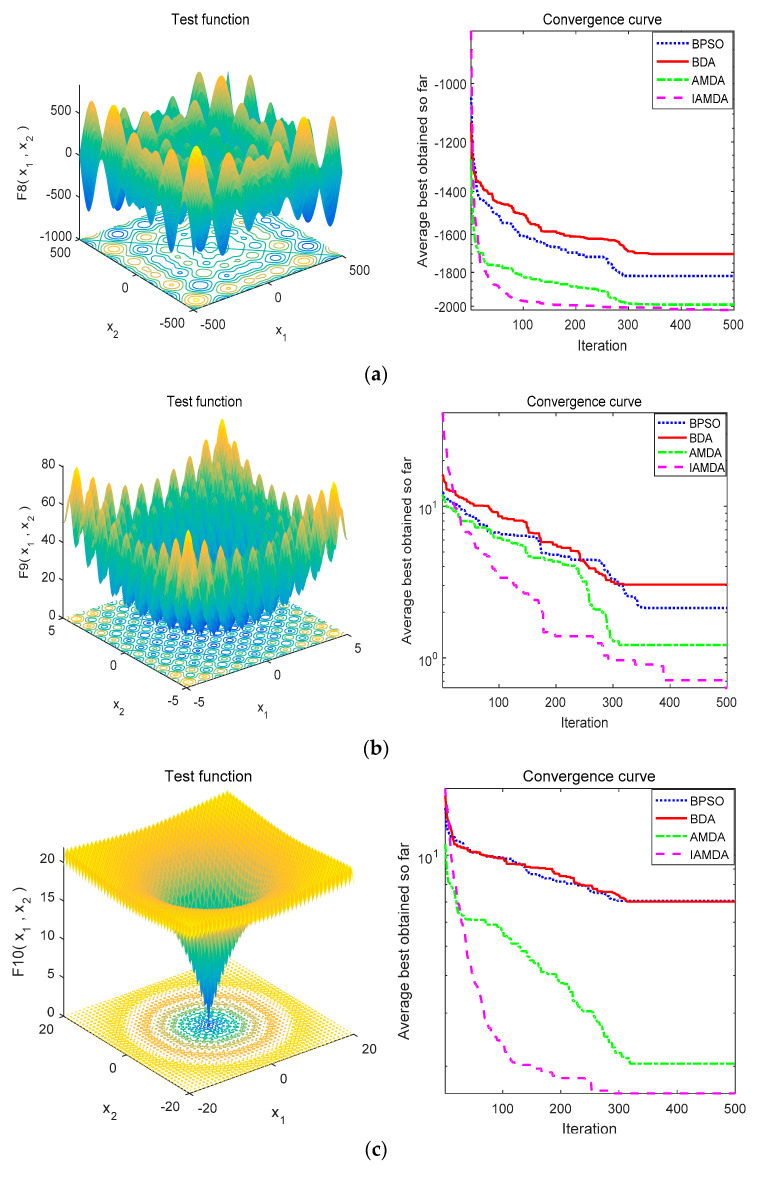

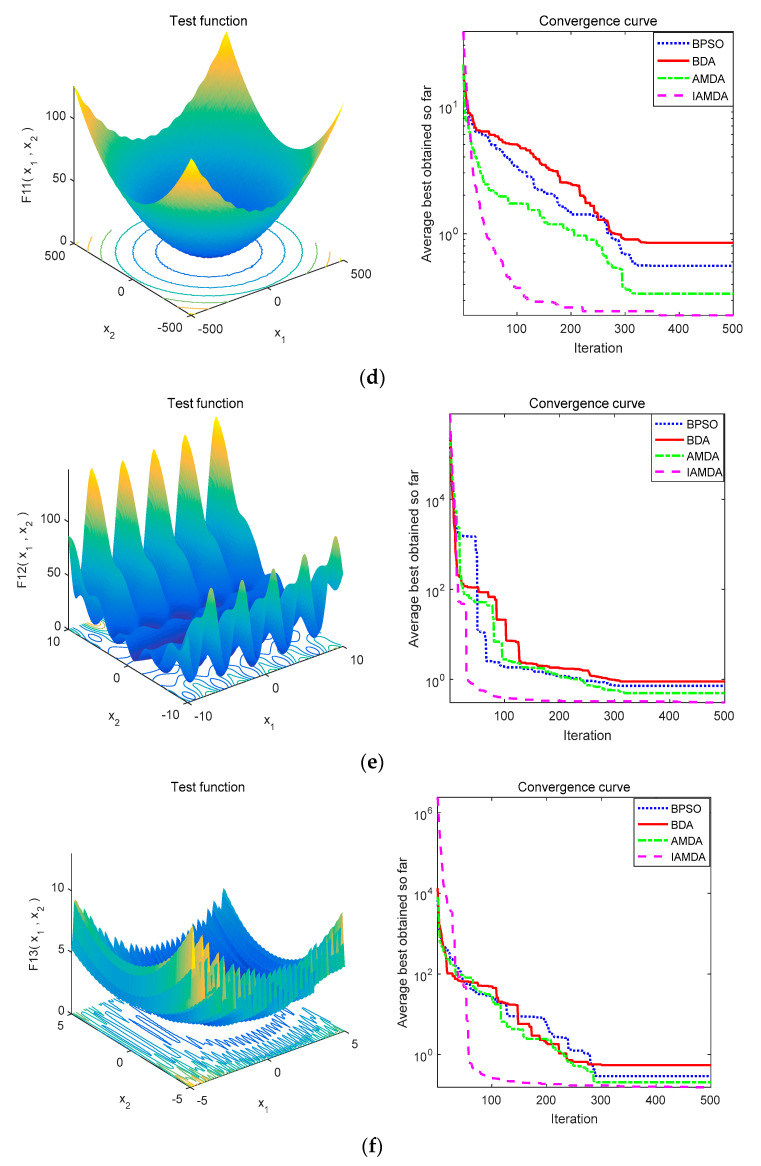
Convergence curve of IAMDA, AMDA, BDA, and BPSO on multimodal functions. (**a**) *f*_8_. (**b**) *f*_9_. (**c**) *f*_10_. (**d**) *f*_11_. (**e**) *f*_12_. (**f**) *f*_13_.

Figure 8 represents the convergence curves of the above four algorithms on different unimodal functions and Figure 9 shows the convergence curves of the above algorithms on various multimodal functions. Table 4 lists the average, median values, and standard deviation of IAMDA, AMDA BDA, and BPSO while testing the benchmark functions.

Figure 8 and Figure 9, respectively, represent the average convergence curves of the four algorithms on 7 unimodal functions and 6 multimodal functions after performing 30 experiments. The convergence curves in Figure 8 and Figure 9 indicate that the convergence speed of IAMDA is significantly faster than that of the other three algorithms. For example, from Figure 8e, Figure 9a,e,f, it can be observed that the convergence of IAMDA can reach the optimal value at about 100 iterations.

In order to test whether IAMDA is statistically significant compared to other algorithms, statistical student’s *t*-test [37] has been performed. The *t* value can be calculated by the following formula:

(17) $$t=\frac{\bar{X_{1}}-\bar{X_{2}}}{\sqrt{\left(SD_{1}^{2}/(n_{1}-1))+(SD_{2}^{2}/(n_{2}-1)\right)}}$$

where $\bar{X_{1}}$, $SD_{1}$, and $n_{1}$ represent the mean value, standard deviation, and size of the first sample (AMDA, or BDA, or BPSO), respectively; $\bar{X_{2}}$, $SD_{2}$, and $n_{2}$ indicate the mean value, standard deviation, and size of the second sample (IAMDA), respectively. In this work, $n_{1}$ = $n_{2}$ = $Dim_{dragonfly}$. The positive *t* value means that IAMDA has better solutions compared to AMDA (or BDA or BPSO). The negative *t* value means that AMDA (or BDA or BPSO) produced better solutions than IAMDA. In our study, the confidence interval has been set at 95% which indicates *t*_0.05_ = 1.96. When *t* > 1.96, the difference between two samples is significant and IAMDA is superior to AMDA (or BDA or BPSO). When *t* < −1.96, AMDA (or BDA or BPSO) is superior to IAMDA.

The *t* values calculated by Equation (17) over the selected 13 benchmark functions are presented in Table 5 and Table 6. In the presented tables, ‘N.S.’ represents ‘Not Significant’, which means that the compared algorithms do not differ from each other significantly.

Note that all the data in Table 4 are average values over 30 experiments. From the experimental results in Table 4, it can be analyzed that as compared to AMDA, BDA, and BPSO, IAMDA has obvious advantages in the optimization results of the 13 standard test functions. Judging from Table 5, IAMDA can find the optimal solutions in most cases of unimodal functions, which means that IAMDA has better exploitation capability. Additionally, according to the *t* values calculated in Table 6, IAMDA and AMDA exhibit similar exploration capability and have better performance than BDA and BPSO on multimodal functions. In brief, IAMDA has better exploitation and exploration capability. This proves that the introduction of the coefficient *k* in the angle modulation mechanism is beneficial for improving the convergence accuracy of the algorithm. Hence, according to the average convergence curves in Figure 8 and Figure 9, and test data in Table 4, Table 5 and Table 6, it can be concluded that IAMDA outperforms the AMDA, BDA, and BPSO.

### 4.3. Zero-One Knapsack Problems

The 0-1 knapsack problem is one of combinatorial optimization problems, which means the time complexity of solving the knapsack problem grows very fast as the scale of the problem grows. Because of its complexity, the 0-1 knapsack problem has extremely crucial applications in number theory research, along with certain practical applications like cryptography [38], project selection [39], and feature selection [40,41,42]. It is a procedure of giving *n* items, and each item has two attributes, namely weight *w*_i_ and profit *p_i_*. Capacity *C* indicates the maximum weight of the knapsack, and *x_i_* represents whether the items *i* can be included in the knapsack or not. The target of 0-1 knapsack problem is to maximize the profit of the items in the knapsack and make the overall weights less than or equal to the knapsack capacity. The zero-one problem can be mathematically modeled as follows [43]:

(18) $$\max f(p)=\sum_{i=1}^{N}p_{i}x_{i}$$

(19) $$s.t.\{\begin{array}{c} f(w)=\sum_{i=1}^{N}w_{i}x_{i}\leq C\\ x_{i}=0,1(i=1,2,...,N) \end{array}$$

Since the procedure of the 0-1 knapsack problem is essentially a binary optimization process, binary heuristic algorithms such as BPSO and BDA are required to solve the 0-1 knapsack problems. The following tables highlight 12 classic 0-1 knapsack problems, including five classic 0-1 knapsack problems *k*_1_–*k*_5_ [44] listed in Table 7 and seven high-dimensional 0-1 knapsack problems *k*_6_–*k*_12_ [45] listed in Table 8. The larger the problem dimension, the greater the computational complexity and the longer the execution time. In the tables, ’D’ indicates the dimension of a knapsack problem, ’*w*’ and ‘*p*’ represent the weight and profit of each object, respectively. ‘*C*’ denotes the capacity of a knapsack, ‘Opt’ shows the optimal value and ‘Total values’ in Table 6 represents overall profits of all items. Table 9 shows the best, worst, and average solutions for 0-1 knapsack problems. Additionally, the average calculation time and the standard deviation (SD) are listed. Table 10 lists the *p* values of the Wilcoxon ranksum test over the seven high-dimensional knapsack problems, ‘N.S.’ represents ‘not significant’, which means that the compared algorithms do not differ from each other significantly.

Table 9 presents the test results of the four algorithms after performing 30 experiments. It can be observed that for *k*_1_ and *k*_2_, all the four algorithms can find the optimal solution. Whereas for *k*_3_–*k*_12_, IAMDA and AMDA can always find better results in less computation time, suggesting the strong global optimization capabilities and computational robustness of IAMDA and AMDA in binary spaces. Additionally, it can be observed that the higher the dimensionality of the 0-1 knapsack problem, the more obvious the advantages of IAMDA and AMDA. Moreover, as compared to AMDA, the standard deviation of IAMDA is much smaller, which suggests that IAMDA is more stable and effective than AMDA for solving the 0-1 knapsack problems. Besides, the *p* values listed in Table 10 prove that IAMDA and AMDA outperform BDA and BPSO in solving large-scale knapsack problems.

**Figure 10:**
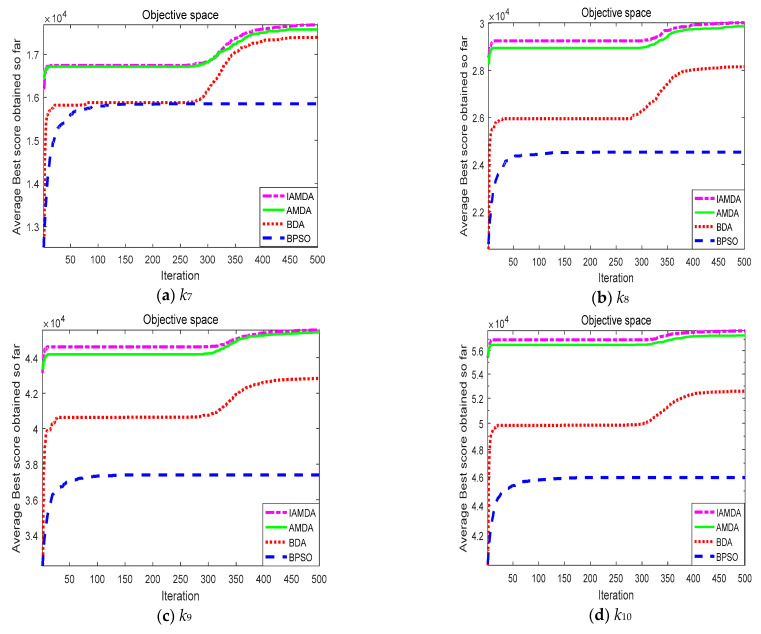

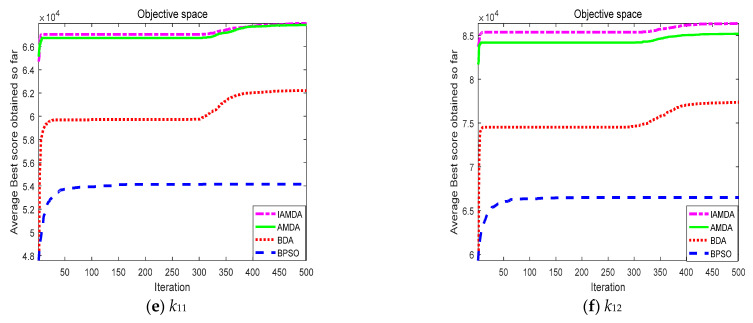
Average convergence curves of IAMDA, AMDA, BDA, and BPSO on some selected large-scale problems over 30 independent runs: (**a**) *k*_7_. (**b**) *k*_8_. (**c**) *k*_9_. (**d**) *k*_10_. (**e**) *k*_11_. (**f**) *k*_12_.

Figure 10 shows the average convergence curves of the four algorithms on the selected large-scale problems in 30 independent runs. As denoted in the figure, (i) the purple curve representing IAMDA is always on the top of the other curves and the effect becomes more obvious with the increasing problem dimension; (ii) the red and blue curves representing BDA and BPSO are slowly climbing, or even stagnating. In other words, IMADA has the strongest convergence, while BDA and BPSO converge prematurely to solve large-scale testing problems.

**Figure 11:**
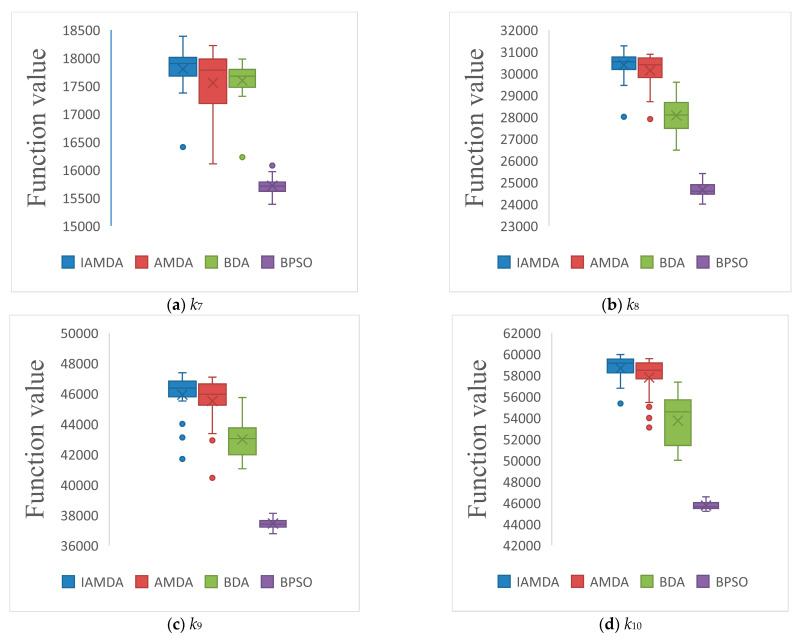

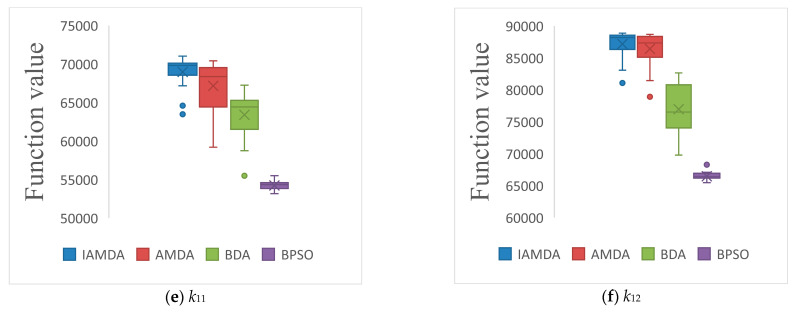
The box plots of IAMDA, AMDA, BDA, and BPSO on some selected large-scale problems. (**a**) *k*_7_. (**b**) *k*_8_. (**c**) *k*_9_. (**d**) *k*_10_. (**e**) *k*_11_. (**f**) *k*_12_.

Figure 11 depicts the distribution of the results of the knapsack problem obtained by the four algorithms. As shown in the figure, (i) the solution produced by IAMDA always give better results, and the results vary within a confined range, thus producing a smaller variance; (ii) diversity of the results produced by BDA is the best; and (iii) in the majority of the problems, many of the results produced by IAMDA are as applicable as those produced by AMDA; however, IAMDA has a smaller variance than AMDA.

**Figure 12:**
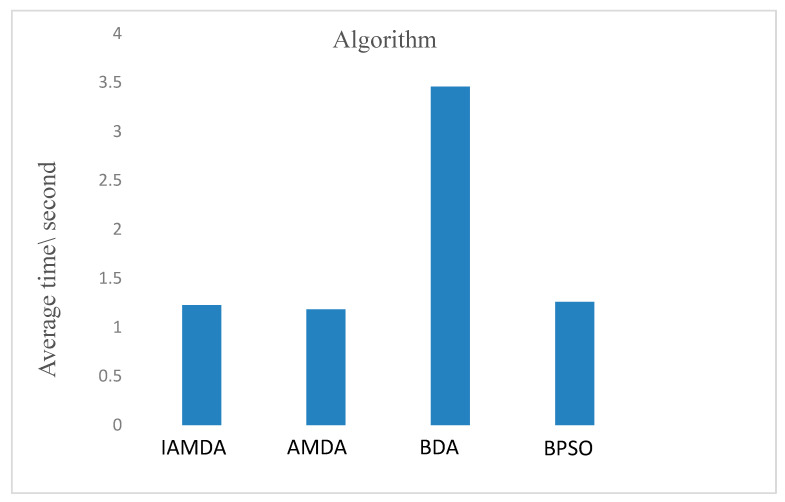
Average computational time of IAMDA, AMDA, BDA, and BPSO on some selected large-scale problems *k*_7_–*k*_12_ over 30 independent runs.

Figure 12 indicates the average computational time of the above algorithms on the selected *k*_1_–*k*_12_ knapsack problems. It can be noted from the bar diagram that (i) the average computational time of AMDA is the least, and (ii) the computational time of IAMDA, AMDA, and BPSO are similar, and all are significantly less than the calculation time of BDA.

It can be summarized from the above simulation results that when IAMDA solves the 0-1 knapsack problems, it decreases the computational time while ensuring the accuracy of the solution. IAMDA performs well on both low-dimensional as well as high-dimensional problems. Moreover, it has a smaller variance than AMDA and the original BDA, indicating better robustness of IAMDA.

## 5. Conclusions

To make the dragonfly algorithm work efficiently in the binary space, this paper applies an angle modulation mechanism to the dragonfly algorithm. AMDA uses the trigonometric function to generate bit strings corresponding to the binary problem solutions instead of directly running on the high-dimensional binary spaces. Thus, AMDA can significantly reduce the computational cost as compared to the traditional BDA using transfer functions. However, AMDA also has some limitations such as poor algorithm stability and slow convergence speed due to the lack of control on the vertical displacement of the cosine part in the generating function.

To deal with the limitations, this paper proposes an improved angle modulated dragonfly algorithm (IAMDA). Based on AMDA, one more coefficient is added to adjust the vertical displacement of the cosine part in the original generating function. From the test results of unimodal and multimodal benchmark functions and 12 low-dimensional and high-dimensional zero-one knapsack problems, it can be concluded that IAMDA outperforms AMDA, BDA, and BPSO in terms of stability, convergence rate, and quality of the solution. Additionally, it significantly reduces the computational time as compared to BDA. For future advancements, our studies may include multidimensional zero-one knapsack problems, multi-objective optimization problems, and so on. Furthermore, our research will be applied to practical applications such as feature selection and antenna topology optimization.

## Figures and Tables

**Table 1:** Initial parameters of IAMDA, AMDA, BDA, and BPSO.

Algorithms	Parameters	Values
IAMDA	Number of dragonflies	30
	(*a*, *b*, *c*, *d*, *k*)	[−1,1]
	Max iteration	500
	Stopping criterion	Max iteration
AMDA	Number of dragonflies	30
	(*a*, *b*, *c*, *d*)	[−1,1]
	Max iteration	500
	Stopping criterion	Max iteration
BDA	Number of dragonflies	30
	Max iteration	500
	Stopping criterion	Max iteration
BPSO	Number of particles	30
	*C*_1_, *C*_2_	2
	*w*	Decreased linearly from 0.9 to 0.4
	Max velocity	0.6
	Max iteration	500
	Stopping criterion	Max iteration

**Table 2:** Unimodal benchmark functions.

Function	Expression	*n*	Range	f_min_
Sphere	$f_{1}(x)={\sum_{i=1}^{n}x}_{i}$	5	[−100,100]	0
Schwefel 2.22	$f_{2}(x)=\sum_{i=1}^{n}\left|x_{i}\right|+\prod_{i=1}^{n}\left|x_{i}\right|$	5	[−10,10]	0
Schwefel 1.2	$f_{3}(x)=\sum_{i=1}^{n}(\sum_{j=1}^{i}x_{j}{)}^{2}$	5	[−100,100]	0
Schwefel 2.21	$f_{4}(x)=\{\left|x_{i}\right|,1\leq i\leq n\}$	5	[−100,100]	0
Rosenbrock	$f_{5}(x)=\sum_{i=1}^{n-1}\left[100(x_{i+1}-x_{i}{}^{2})+{\left(x_{i}-1\right)}^{2}\right]$	5	[−30,30]	0
Step	$f_{6}(x)=\sum_{i=1}^{n}\left([x_{i}+0.5\right]{)}^{2}$	5	[−100,100]	0
Quartic	$f_{7}(x)=ix_{i}{}^{4}+random[0,1)$	5	[−1.28,1.28]	0

**Table 3:** Multimodal benchmark functions.

Function	Expression	*n*	Range	f_min_
Schwefel	$f_{8}(x)=\sum_{i=1}^{n}-x_{i}\sin(\sqrt{\left|x_{i}\right|})$	5	[−500,500]	−418.9829 × 5
Rastrigrin	$f_{9}(x)=\sum_{i=1}^{n}\left[x_{i}{}^{2}-10\cos(2\pi x_{i})+10\right]$	5	[−5.12,5.12]	0
Ackley	$\begin{array}{l} f_{10}(x)=-20\exp(-0.2\sqrt{\frac{1}{n}\sum_{i=1}^{n}x_{i}{}^{2}})\\ -\exp(\frac{1}{n}\sum_{i=1}^{n}\cos(2\pi x_{i}))+20+e \end{array}$	5	[−32.32]	0
Griewank	$\begin{array}{l} f_{11}(x)=\frac{1}{4000}\sum_{i=1}^{n}x_{i}{}^{2}\\ -\prod_{i=1}^{n}\cos(\frac{x_{i}}{\sqrt{i}})+1 \end{array}$	5	[−600,600]	0
Penalty#	$\begin{array}{l} f_{12}(x)=\frac{\pi }{n}\{10\sin(\pi y_{1})+\sum_{i=1}^{n-1}(y_{i}-1{)}^{2}[1\\ +\sin(\pi y_{i}+1)]+{\left(y_{n}-1\right)}^{2}\}\\ +\sum_{i=1}^{n}u(x_{i},10,100,4)\}\\ yi=1+\frac{x_{i}+1}{4}\\ u(x_{i},a,k,m)=\{\begin{array}{c} k{\left(x_{i}-a\right)}^{m},x_{i}>a\\ 0,-a<x_{i}<a\\ k{\left(-x_{i}-a\right)}^{m},x_{i}<-a \end{array} \end{array}$	5	[−50,50]	0
Penalized 1.2	$\begin{array}{l} f_{13}(x)=0.1\{\sin^{2}(3\pi x_{1})+\sum_{i=1}^{n}(x_{i}-1{)}^{2}[1\\ +\sin^{2}(3\pi x_{i}+1)]+{\left(x_{n}-1\right)}^{2}[1+\sin^{2}(2\pi x_{n})]\}\\ +\sum_{i=1}^{n}u(x_{i},5,100,4) \end{array}$	5	[−50,50]	0

**Table 4:** Performance comparison among IAMDA, AMDA, BDA, and BPSO on unimodal benchmark functions and multimodal benchmark functions.

*f*	Metric	IAMDA	AMDA	BDA	BPSO
*f*_1_	Mean	**0.2244**	1.2797	1.8412	1.5942
	SD	**0.1599**	0.9732	1.6625	1.3609
	Med	**0.1510**	0.6655	1.6669	1.3278
	Rank	1	2	4	3
*f*_2_	Mean	**0.0676**	0.1751	0.1883	0.2137
	SD	**0.0216**	0.0913	0.1004	0.0882
	Med	**0.0585**	0.1423	0.2765	0.2702
	Rank	1	2	3	4
*f*_3_	Mean	**3.4279**	15.1108	19.8865	21.8408
	SD	**1.9467**	14.1566	22.7839	11.6904
	Med	**2.4599**	18.7306	19.1034	23.0999
	Rank	1	2	3	4
*f*_4_	Mean	**0.3383**	0.6840	0.8205	0.8701
	SD	**0.0988**	0.4461	0.3266	0.3559
	Med	**0.3446**	0.6906	0.9330	0.9568
	Rank	1	2	3	4
*f*_5_	Mean	**22.6485**	36.2572	82.5599	133.7547
	SD	**3.0791**	10.9330	11.4133	9.5546
	Med	**20.4659**	30.0536	71.9376	124.0569
	Rank	1	2	3	4
*f*_6_	Mean	**1.0166**	2.2309	3.0771	8.9318
	SD	**0.5524**	11.0604	9.7121	13.7554
	Med	**1.7111**	3.6660	3.1051	11.9169
	Rank	1	2	3	4
*f*_7_	Mean	**0.0179**	0.0186	0.0387	0.0309
	SD	**0.0119**	0.0156	0.0235	0.0216
	Med	**0.0163**	0.0182	0.0327	0.0335
	Rank	1	2	4	3
*f*_8_	Mean	**−2.0236e+03**	−1.9898e+03	−1.7002e+03	−1.8200e+03
	SD	**53.4828**	170.8601	147.8324	101.7015
	Med	**−2.0207e+03**	−1.8977e+03	−1.6972e+03	−1.8307e+03
	Rank	1	2	4	3
*f*_9_	Mean	**0.6359**	1.2144	3.0472	2.1303
	SD	**1.7575**	5.5272	3.7159	5.0115
	Med	**0.7802**	0.8932	3.5783	2.3890
	Rank	1	2	4	3
*f*_10_	Mean	**1.6269**	2.0406	7.0218	7.0607
	SD	**0.7271**	2.4153	2.5491	2.4290
	Med	**1.4469**	2.3801	6.7427	7.4276
	Rank	1	2	3	4
*f*_11_	Mean	**0.2291**	0.3381	0.8498	0.5594
	SD	**0.2448**	1.2435	1.6054	1.1212
	Med	**0.2821**	0.4894	0.7234	0.4527
	Rank	1	2	4	3
*f*_12_	Mean	**0.3092**	0.5014	0.8959	0.7153
	SD	**0.1880**	0.3313	0.5359	0.4502
	Med	**0.3947**	0.4381	0.8416	0.7652
	Rank	1	2	4	3
*f*_13_	Mean	**0.1547**	0.2057	0.5492	0.2908
	SD	**0.0675**	0.7528	2.2227	1.2097
	Med	**0.1654**	0.2089	0.5847	0.1755
	Rank	1	2	4	3

**Table 5:** Results of *t*-test for IAMDA against other three algorithms on unimodal benchmark functions with 30 independent runs.

*f*	AMDA and IAMDA		BDA and IAMDA		BPSO and IAMDA	
	*t*	Sig.	*t*	Sig.	*t*	Sig.
*f*_1_	9.2046	IAMDA	8.3274	IAMDA	8.5994	IAMDA
*f*_2_	9.8566	IAMDA	10.1103	IAMDA	13.8404	IAMDA
*f*_3_	7.0330	IAMDA	6.1916	IAMDA	13.3650	IAMDA
*f*_4_	6.5086	IAMDA	12.1566	IAMDA	12.3855	IAMDA
*f*_5_	10.3097	IAMDA	43.5972	IAMDA	95.2107	IAMDA
*f*_6_	0.9433	N.S.	1.9721	IAMDA	4.9460	IAMDA
*f*_7_	0.3069	N.S.	6.7927	IAMDA	4.5347	IAMDA

**Table 6:** Results of *t*-test for IAMDA against other three algorithms on multimodal benchmark functions with 30 independent runs.

*f*	AMDA and IAMDA		BDA and IAMDA		BPSO and IAMDA	
	*t*	Sig.	*t*	Sig.	*t*	Sig.
*f*_8_	1.9640	IAMDA	17.6961	IAMDA	15.2422	IAMDA
*f*_9_	0.8580	N.S.	5.0462	IAMDA	2.4206	IAMDA
*f*_10_	1.4109	N.S.	17.5076	IAMDA	18.4356	IAMDA
*f*_11_	0.7398	N.S.	3.2879	IAMDA	2.4759	IAMDA
*f*_12_	4.3404	IAMDA	8.8868	IAMDA	7.1604	IAMDA
*f*_13_	0.5805	N.S.	1.9661	IAMDA	2.9663	IAMDA

**Table 7:** Related parameters of five classic 0-1 knapsack problems.

No.	D	Parameter (*w*, *p*, *C*)	Opt
*k*_1_	10	*w* = (95,4,60,32,23,72,80,62,65,46); *p* = (55,10,47,5,4,50,8,61,85,87); *C* = 269	295
*k*_2_	20	*w* = (92,4,43,83,84,68,92,82,6,44,32,18,56,83,25,96,70,48,14,58); *p* = (44,46,90,72,91,40,75,35,8,54,78,40,77,15,61,17,75,29,75,63); *C* = 878	1024
*k*_3_	50	*w* = (80,82,85,70,72,70,66,50,55,25,50,55,40,48,59,32,22,60,30, 32,40,38,35,32,25,28,30,22,50,30,45,30,60,50,20,65,20,25,30, 10,20,25,15,10,10,10,4,4,2,1); *p* = (220,208,198,192,180,180,165,162,160,158,155,130,125, 122,120,118,115,110,105,101,100,100,98,96,95,90,88,82,80,77,75,7,72,70,69,66,65,63,60,58,56,50,30,20,15, 0,8,5,3,1); *C* = 1000	3103
*k*_4_	80	*w* = (40, 27,5,21,51, 16, 42, 18, 52, 28, 57, 34, 44, 43,52,55,53,42, 47, 56,57,44, 16,2, 12, 9, 40, 23, 56, 3, 39,16, 54, 36, 52,5,53, 48, 23, 47, 41, 49, 22, 42, 10, 16, 53, 58, 40, 1,43,56,40,32,44,35, 37, 45, 52, 56, 40, 2, 23,49, 50, 26, 11,35, 32, 34, 58, 6, 52,26,31, 23, 4, 52, 53, 19); *p* = (199,194,193,191,189,178,174,169,164,164,161,158,157, 154,152,152,149,142,131,125,124,124,124,122,119,116,114,113,111,110,109,100,97,94,91,82,82,81,80,80,80,79,77,76,74, 72, 71, 70, 69,68, 65, 65, 61, 56, 55, 54, 53, 47, 47, 46, 41, 36, 34, 32, 32,30, 29, 29, 26, 25, 23, 22, 20, 11, 10, 9,5,4,3, 1); *C* = 1173	5183
*k*_5_	100	*w* = (54, 95, 36, 18,4, 71,83, 16, 27, 84, 88, 45, 94, 64, 14, 80, 4, 23, 75, 36, 90, 20, 77, 32, 58, 6, 14, 86, 84, 59,71, 21, 30, 22, 96, 49, 81, 48, 37, 28, 6, 84,19,55,88,38,51,52,79,55,70,53,64,99,61,86,1,64,32,60,42,45,34,22,49,37,33,1,78,43,85,24,96,32,99,57,23,8,10,74,59,89,95,40,46,65,6,89,84,83,6,19,45, 59, 26, 13, 8, 26, 5, 9); *p* = (297, 295, 293, 292, 291, 289, 284, 284, 283, 283, 281, 280, 279, 277, 276, 275, 273,264, 260, 257, 250, 236, 236, 235, 235, 233, 232, 232, 228, 218, 217, 214, 211, 208, 205, 204, 203, 201, 196, 194,193, 193, 192, 191, 190, 187, 187, 184, 184, 184, 181, 179, 176, 173, 172, 171, 160, 128, 123, 114, 113, 107, 105,101, 100, 100, 99, 98, 97, 94, 94, 93, 91, 80, 74, 73, 72, 63, 63, 62, 61, 60, 56, 53, 52, 50, 48, 46, 40, 40, 35, 28, 22,22, 18, 15, 12,11, 6,5); *C* = 3818;	15,170

**Table 8:** Related parameters of seven randomly generated zero-one knapsack problems.

No.	D	C	Total Values
*k*_6_	200	1948.5	15,132
*k*_7_	300	2793.5	22,498
*k*_8_	500	4863.5	37,519
*k*_9_	800	7440.5	59,791
*k*_10_	1000	9543.5	75,603
*k*_11_	1200	11,267	90,291
*k*_12_	1500	14,335	111,466

**Table 9:** Result comparisons among IAMDA, AMDA, BDA, and BPSO on 0-1 knapsack problems.

No.	Alg.	Best	Worst	Mean	SD	Time
*k*_1_	IAMDA	**295**	**295**	**295**	0	0.2175
	AMDA	**295**	**295**	**295**	0	0.2112
	BDA	**295**	**295**	**295**	0	0.9646
	BPSO	**295**	**295**	**295**	0	**0.0389**
*k*_2_	IAMDA	**1024**	1018	1.0231e+03	2.1981	0.2858
	AMDA	**1024**	1013	1.0226e+03	3.5452	0.2766
	BDA	**1024**	1018	1.0225e+03	2.6656	1.0450
	BPSO	**1024**	**1024**	**1024**	**0**	**0.0618**
*k*_3_	IAMDA	**3076**	**2991**	3.0308e+03	**21.1636**	0.2863
	AMDA	3064	2969	**3.0367e+03**	29.6729	0.2782
	BDA	3074	2970	3.0203e+03	30.0559	1.0461
	BPSO	3074	2957	2.9978e+03	26.6114	**0.1460**
*k*_4_	IAMDA	4991	**4763**	**4.9131e+03**	**66.2322**	0.3929
	AMDA	**5090**	4678	4.8918e+03	115.7004	0.3796
	BDA	5041	4705	4.8880e+03	105.7243	1.2035
	BPSO	4695	4348	4.4823e+03	99.1591	**0.2175**
*k*_5_	IAMDA	14,965	**14,261**	**1.4631e+04**	**166.4238**	0.3979
	AMDA	**15,010**	14,155	1.4626e+04	220.8765	0.3803
	BDA	14,986	14,149	1.4611e+04	208.1295	1.2696
	BPSO	13,986	13,324	1.3595e+04	188.0612	**0.2488**
*k*_6_	IAMDA	**1.3075e+04**	**1.2300e+04**	**1.2632e+04**	**207.7428**	0.4770
	AMDA	1.2801e+04	1.1921e+04	1.2498e+04	211.4083	**0.4631**
	BDA	1.2820e+04	1.1501e+04	1.2316e+04	315.2521	1.6793
	BPSO	1.1640e+04	1.0951e+04	1.1174e+04	213.5035	0.6185
*k*_7_	IAMDA	**1.8386e+04**	**1.6408e+04**	**1.7800e+04**	413.3773	0.6500
	AMDA	1.8220e+04	1.6107e+04	1.7595e+04	594.7544	**0.6137**
	BDA	1.7979e+04	1.6227e+04	1.7554e+04	370.3743	2.8821
	BPSO	1.6084e+04	1.5385e+04	1.5717e+04	**181.8523**	0.8482
*k*_8_	IAMDA	**3.1266e+04**	**2.8010e+04**	**3.0387e+04**	713.8203	0.9952
	AMDA	3.0763e+04	2.7902e+04	3.0134e+04	816.0871	**0.9457**
	BDA	2.9598e+04	2.6478e+04	2.8067e+04	848.2838	3.6978
	BPSO	2.5404e+04	2.3997e+04	2.4656e+04	**328.1345**	1.3125
*k*_9_	IAMDA	**4.7364e+04**	**4.1702e+04**	**4.5928e+04**	1.4190e+03	1.7125
	AMDA	4.7078e+04	4.0453e+04	4.5502e+04	1.6564e+03	**1.7014**
	BDA	4.5734e+04	4.1055e+04	4.2988e+04	1.2721e+03	5.3235
	BPSO	3.8119e+04	3.6775e+04	3.7448e+04	**355.7410**	2.0791
*k*_10_	IAMDA	**5.9952e+04**	5.5355e+04	5.8646e+04	1.3125e+03	2.5047
	AMDA	5.9566e+04	5.3099e+04	5.7783e+04	1.8917e+03	**2.5023**
	BDA	5.7356e+04	5.0011e+04	5.3727e+04	2.3538e+03	6.2211
	BPSO	4.6572e+04	4.5209e+04	4.5749e+04	**362.8049**	2.6863
*k*_11_	IAMDA	**7.1022e+04**	**6.3479e+04**	**6.8977e+04**	1.9784e+03	3.2814
	AMDA	7.0417e+04	5.9200e+04	6.7161e+04	3.0546e+03	3.0616
	BDA	6.7241e+04	5.5492e+04	6.3396e+04	3.0978e+03	7.6517
	BPSO	5.5506e+04	5.3168e+04	5.4227e+04	**552.3881**	**3.0838**
*k*_12_	IAMDA	**8.8872e+04**	**8.1067e+04**	**8.7179e+04**	2.1245e+03	3.5053
	AMDA	8.8691e+04	7.8917e+04	8.6422e+04	2.5499e+03	**3.3711**
	BDA	8.2644e+04	6.9772e+04	7.6970e+04	3.9042e+03	8.5147
	BPSO	6.7097e+04	6.5470e+04	6.6496e+04	**648.1773**	3.7690

**Table 10:** *p*-values of the Wilcoxon ranksum test on large-scale knapsack problems.

*f*	AMDA and IAMDA		BDA and IAMDA		BPSO and IAMDA	
	*t*	Sig.	*t*	Sig.	*t*	Sig.
*k*_6_	0.0709	N.S.	0.0937	IAMDA	6.7956e-08	IAMDA
*k*_7_	0.2085	N.S.	0.0066	IAMDA	6.7956e-08	IAMDA
*k*_8_	0.3793	N.S.	4.5390e-07	IAMDA	6.7956e-08	IAMDA
*k*_9_	0.2393	N.S.	5.8736e-07	IAMDA	6.7956e-08	IAMDA
*k*_10_	0.0409	IAMDA	3.4156e-07	IAMDA	6.7956e-08	IAMDA
*k*_11_	0.0155	IAMDA	2.6898e-06	IAMDA	6.7956e-08	IAMDA
*k*_12_	0.1636	N.S.	1.2346e-07	IAMDA	6.7956e-08	IAMDA

## Data Availability

Data is contained within the article.
